# Dental Education. No. I

**Published:** 1876-12

**Authors:** J. Clark Scott


### DENTAL EDUCATION. NO. I.

BY J. CLARK SCOTT, D. D. S.

*Advice to young men who desire to enter the Dental Profession.*

The profession and practice of dental science afford an almost unlimited field for honorable and lucrative occupation, which will continue, without doubt, while the race of man continues. The man who, with all proper qualifications and honest purpose devotes his life assiduously and unvaryingly to the care of the teeth, in all their relations, both as to the preservation and restoration of the health and usefulness of those important organs can confer as much good upon mankind, as in any pursuit which is open to his career. The profession and practice of dentistry, in common with all other vocations, has its pleasant and unpleasant parts; its bitters and sweets; it is not an undisturbed course of the agreeable; far from it. Yet that which the dentist has to do is usually patent before him; his duties are distinctly indicated, and if he never deviates from duty, or leaves undone that which he should do, he will find in his consciousness of rectitude a self-congratulation that will more than compensate him for the unpleasant and annoying scenes he will be called upon to encounter.

The first thing to be determined by a young man who thinks of choosing the profession of dentistry should be his qualifications, mental and artistical. That some are better qualified by native ability to become skillful, and to succeed in that particular calling than are others is true. Every man has a capacity for some special duties that will enable him to succeed better in that special line than he could in any other; and every thing concurs to show that the want of success in so many men is the result of their having chosen the wrong business.

There are good, medium and indifferent capacities engaged in every calling of life; and in proportion to these degrees of fitness will be the success, all things else considered: Mere mechanical genius and the requisite qualities that fit one for a surgeon will not, of themselves, make a good dentist; and yet these are indispensable to success; but added to these must be all the nobler qualities of the mind, such as patience, strict and invariable temperance, the sense of justice and moral accountability, courtesy and forbearance. But justice to the dentist and to his patients will not permit him to compromise any part of his duties on account of any fears of giving pain, or performing any unpleasant duty. Again, aside from mechanical and surgical qualities, the man who would be a dentist must be both a scholar and a gentleman in every sense of the word. To be a gentleman truly is to possess all the qualities of a good man, and to wear in every expression and manifestation of demeanor the agreeable and the pleasant.

The boor in manners and intercourse, though he might possess all the other qualities requisite for a successful dentist, will never succeed; his very repulsiveness will compromise all hopes of gaining either friends or wealth.

Dental operations are always dreaded, and to be in the hands of an operator who handles the mouth as if he were splitting rails or sawing wood, and whose address and manners are any thing but affable, makes the sitting one not likely to be repeated under the same circumstances. All these as preliminary considerations, are to be duly entertained and wisely disposed of as the first step towards entering upon the responsibilities of the professional dentist.

If, upon due and careful examination, any one can satisfy himself he is deficient in any of these conditions or qualities, and can not make up his mind to be governed by the principles here laid down, he is looking in the wrong direction, and should at once and forever abandon the thought of becoming a dentist; he is better fitted for some other calling and should at once seek it.

But having the qualifications above indicated, and having decided to take up the life of a dentist, I beg you—permit me to tender some hints that may be useful to you in after life. First, if you have chosen the dental profession alone because you think you can make money quicker and with less labor than you could at something else, your resolve is dishonorable in so far as that idea is concerned, and you should endeavor to be controlled by higher motives. But if you become a dentist you should make money by the business, and you will if the right course is pursued. Again, have you conceived the idea that you can enter the profession with less sacrifice of time and with less capital than you could that of medicine, law, trade, or the mechanical department, or farming? Then, again, are you wrong. Your capital to start on must be, in the first place, two years at least of your time devoted to study and instructions under a reputable practitioner, including two full courses of lectures in a reputable dental college, at the expiration of which time you are given a diploma; that is providing you are found by examination to be worthy. You will then require an outfit of from five hundred to a thousand dollars to furnish you with all that is needed to prosecute your chosen profession successfully.

The intelligence of the community has now arrived at such a standard and the respectability of the profession has been planted on such a basis that any thing short of this will not give you a standing that will culminate in success.

The days when men could leave their benches or the plow and become dentists by collecting a few instruments, mostly of their own construction, and with a book of tin foil and a few pivot teeth, take the "circuit," pocketing their fees by the handful have passed by forever. With such men whatever of proficiency or skill in operating has been acquired has always been done at a fearful sacrifice of the human teeth by literally using the mouths of patients as so many apprentice shops in which to learn a trade, and making the territory travelled over an unsafe course for the dentist to appear upon afterwards. These days we repeat are past; though much of the early dentistry was done in this way. People now require an educated and competent class of men to take charge of their teeth, and as this class of men come upon the stage, dentistry grows in magnitude and importance, and its remuneration grows proportionately. People pay cheerfully and liberally for that which contributes to their health and comfort, and in dental science especially they are beginning to recognize where their interest lies. If then you would make yourself reputable and make money too, place yourself in the right attitude before the world. In entering upon the active duties of your profession, you will meet with many annoyances that you do not now think of, and in the prosecution of labors you will encounter many perplexities; but whatever befalls you, let your motto be this, do what is right to be done or do nothing. Do not perform an operation for the sake of increasing your receipts, while your better judgment tells you it should not be done; or because your patient requsts it. You know what is right, if you are suited for the place. Your patients can not always know. You are to stand firmly upon your sense of duty. You will be asked a great many questions by some persons, all of which it is your duty to answer candidly and truthfully, and all that it is proper for the patient to know. In this your own reputation will to some extent be at stake; but your sense of duty will prevent you from misleading those who confide in you or making promises that can not be realized.

You will also find persons who will assume the dictatorial, and tell you what to do or not to do. When you meet with such cases, state straight through what you must do, if you operate at all, In all such cases these positions are taken by persons who are ignorant of the nature or intention of dental operations, but who are yet anxious to be benefited by your art. I have never found it difficult to overcome all such fears by necessary explanations. Almost all persons are disposed to be reasonable, if they are approached in the right way. When you have given your reasons for what you propose to do, backed by firmness, self-confidence and coolness, you will in ninty-nine cases in the hundred secure the lasting confidence of your patient, and to yourself an approving conscience, worth more to you than gold.

In all your operations be calm and self-possessed, by all means never suffer yourself to be in a hurry or in haste, no matter how many patients you have in waiting. Nothing embarrasses an operator so much as to be compelled to feel in a hurry, and no good work can ever be done when done in a hurry, and in nothing is this more true than in operations on the teeth. I have never been able to make a good filling in my life when I have suffered myself to be hurried. It has sometimes been the case, when I have had several persons from the country waiting for my services. Upon filling teeth, you must work with great deliberation, and a steady hand, and if it takes five or six hours to fill one cavity, use five or six hours and charge what it is worth. If you save your patient's tooth, he gets the best of the bargain after all, and will always thank you for services rendered. The idea of filling from ten to twenty cavities in a single day is supremely absurd. No man every did it and made even respectable work. I have been able to fill on an average about five cavities a day. I never allow myself to attempt more than this number unless the cavities are very small.

I just now call to mind a dentist who filled twelve cavities before breakfast and I refilled the same cavities eight months afterward, and the time I consumed was about two and a half days. There are many petty annoyances presented almost daily at the dentist's office, which will tax your patience and philosophy sorely and require coolness and deliberation on your part. The mouth is not manipulated as wood or iron. The difficulties to be encountered are various and complicated. The teeth are living sensitive organs, and require delicate manipulating, which, with the nervousness and fears of the patient, often tax the patience and endurance of the operator to the utmost. These perplexities you must be prepared to meet, and if you can not command your philosophy so as to deport yourself firmly and composedly through them, you do not possess all the qualifications for a dentist, and you will do better in some other sphere of business. The extraction of teeth is a part of your duties that will sometimes command all of your resources. One thing let me say to you at this point; never say to your patient, "I can extract your tooth in an instant, and you will scarcely feel it." If you so far commit yourself you will occasionly be most sadly mistaken. You will sometimes break a tooth in attempting its extraction, in spite of all your skill and care; and when you do, after having given your patient such assurance, you will have destroyed everything like confidence on his part so effectually, that all your subsequent efforts will be unavailing to regain it. There are teeth so firmly fixed in their sockets and at the same time with such fragile crowns that their removal by the first effortwill be impossible without fracturing them, and when this takes place, there is frequently more pain inflicted than would have been if the tooth had come out, besides greatly increasing the difficulty of the subsequent removing of the roots. It is true that a large majority of all human teeth will be readily removed with forceps by the first effort; but this is only known after the operation, not before. No man, I care not what his experience may have been, can tell by the mere appearance of the crown of a tooth whether it will be easy to extract. There are some appearances of the teeth, and the thickness and configuration of the jaws that can be relied on in a general way, but these often fail us. I have often extracted a tooth with promptness and great satisfaction to myself, and subsequently in attempting to remove another from the same mouth, have found all I could do, and sometimes more too. If you have teeth to extract, go about it firmly and deliberately, and accomplish it the best you can, but you will always succeed the best when you can preserve yourself calmly, using your instruments slowly and steadily. If a failure should occur, it will then be your duty to afford your patient, in an explicit manner, the true reason for the failure.

You will, in that way, seldom fail to satisfy the mind of the sufferer and secure his confidence; a hundred times more so than if you assume, in advance, too much wisdom, which only fastens on the mind of your patrons a conviction of your ignorance and incompetency, which no subsequent course you can adopt will undo. The boasting, self-sufficient fool, never gains the patronage of sensible people. He may for a time inveigle the credulous and unwary; but even with those he soon runs himself out. The world over, the man who arrogates to himself superior knowledge, does but put upon his frontispiece, in brazen relief, the insignia of the ignoramus. A courteous, even and firm course, with perfect self-reliance, is the sign of a successful man.

If you are going to insert artificial teeth, do not let your anxiety to obtain business lead you to over state the case. Do not tell your patients that they can eat as well with artificial teeth as the natural ones. The disappointment that is sure to follow assurance will detract from your reliabilty. Generally the judgment of sensible people will condemn such promises in advance.

Your true course will be to state the case as you know it will come out; if anything, understate it. Upon another matter I wish to advise you, that of professional fees. Cheap dentistry is cheap indeed. My dear brother if you wish to succeed, charge respectable fees; for if your services accomplish what they propose to do, they are worth paying for; and those who are benefited by dental operations never begrudge what it costs them.

There is after all, an inate sense of justice in man, by which he descriminates between right and wrong.

Cheap goods or cheap services, are never sought after by the best patrons; they are in the habit of associating the price with the quality; and the very fact of low prices being named, is synonymous with the little worth of that proposed for sale, and the effect of such a course, if you adopt it, will be to turn away from your office the best patients; or rather the best payers. On the other hand you are not to assume to yourself superiority of ability as a predicate for extra high fees. This will also generally be against you. The public will not be slow to see your mistake, and treat you accordingly. There are men, it is true, who, by their age and reputation can command higher prices than others, but this is often in violation of right. Duty demands that you should charge for your services, prices which will sustain a respectable office, and live respectably at the same time. If you are qualified for the duties of your profession, it has cost you a considerable sum of money to acquire such qualifications. Again; the man who is willing to work for small fees, can neither give time nor labor to his operations which their importance require. The man who would fill a cavity with gold for one dollar must do his work rapidly and consequently imperfectly.

Be honest to your patient and also to yourself—rest assured, success will come.

